# On the strong convergence for weighted sums of negatively superadditive dependent random variables

**DOI:** 10.1186/s13660-017-1530-9

**Published:** 2017-10-27

**Authors:** Bing Meng, Dingcheng Wang, Qunying Wu

**Affiliations:** 1 School of Mathematical Science, University of Electronic Science and Technology of China, Chengdu, Sichuan 611731 P.R. China; 2 College of Science, Guilin University of Technology, Guilin, Guangxi 541004 P.R. China

**Keywords:** 60F15, negatively superadditive dependent random variables, strong convergence, weighted sums

## Abstract

In this article, some strong convergence results for weighted sums of negatively superadditive dependent random variables are studied without assumption of identical distribution. The results not only generalize the corresponding ones of Cai (Metrika 68:323-331, 2008) and Sung (Stat. Pap. 52:447-454, 2011), but also extend and improve the corresponding one of Chen and Sung (Stat. Probab. Lett. 92:45-52, 2014).

## Introduction

Let $\{X_{n}; n\geq1 \}$ be a sequence of random variables defined on a fixed probability space $(\Omega, \mathscr{F}, P )$. We first review the definitions of negatively associated random variables and negatively superadditive dependent (NSD) random variables.

### Definition 1.1

A finite family of random variables $\{X_{i};1\leq i\leq n\}$ is said to be negatively associated (NA) if for every pair of disjoint subsets $A_{1}$ and $A_{2}$ of $\{1,2,\ldots ,n\}$,

1.1 $$ \operatorname{Cov} \bigl(f_{1}(X_{i},i\in A_{1}),f_{2}(X_{j},j\in A_{2}) \bigr) \leq0, $$

whenever $f_{1}$ and $f_{2}$ are coordinatewise nondecreasing functions such that this covariance exists. An infinite family of random variables $\{X_{n};n\geq1\}$ is said to be NA if every finite subfamily is NA.

### Definition 1.2

(Kemperman [4])

A function *ϕ*: $\mathbb{R}^{n}\rightarrow\mathbb{R}$ is called superadditive if $\phi (x\vee y )+\phi (x\wedge y )\geq\phi (x )+\phi (y )$ for all $x,y\in\mathbb{R}^{n}$, where ∨ is for a componentwise maximum and ∧ is for a componentwise minimum.

### Definition 1.3

(Hu [5])

A random vector $X= (X_{1},X_{2},\ldots,X_{n} )$ is said to be NSD if

1.2 $$ E\phi (X_{1},X_{2},\ldots,X_{n} ) \leq E\phi \bigl(X_{1}^{*},X_{2}^{*}, \ldots,X_{n}^{*} \bigr), $$

where $X_{1}^{*},X_{2}^{*},\ldots,X_{n}^{*}$ are independent such that $X_{i}^{*}$ and $X_{i}$ have the same distribution for each *i* and *ϕ* is a superadditive function such that the expectations in (1.2) exist. A sequence of random variables $\{X_{n};n\geq1\}$ is said to be NSD if for every $n\geq1$, $(X_{1},X_{2},\ldots,X_{n} )$ is NSD.

The concept of NA was given by Joag-Dev and Proschan [6], and the concept of NSD was introduced by Hu [5], which was based on the class of superadditive functions. Hu [5] gave an example illustrating that NSD random variables are not necessarily NA, and left an open problem whether NA random variables implies NSD. Christofides and Vaggelatou [7] solved this open problem and showed that NA implies NSD. Thus, it is shown that NSD is much weaker than NA. Because of the wide application of NSD random variables, many authors have studied this concept and obtained some interesting results and applications. For example, we refer to [8–13]. Hence, it is of important significance to extend the limit properties of NA to the case of NSD random variables.

The concept of complete convergence was introduced by Hsu and Robbins [14] as follows. A sequence of random variables $\{X_{n};n\geq1\} $ is said to converge completely to a constant *λ* if

1.3 $$ \sum_{n=1}^{\infty}P\bigl( \vert X_{n}-\lambda \vert >\varepsilon \bigr)< \infty\quad\text{for all }\varepsilon>0. $$

In view of the Borel-Cantelli lemma, the sequence of random variables $\{X_{n};n\geq1\}$ converging completely to a constant *λ* implies $X_{n}\rightarrow\lambda$ almost surely (a.s.). Therefore, the complete convergence of random variables is a very important tool in establishing almost sure convergence. The first results concerning complete convergence for normed sums of random variables were due to Hsu and Robbins (1947) [14] and Erdös (1949) [15], and the obtained results have been extended in several directions by many authors. One can refer to [16–20], etc.

Recently, Cai [1] obtained the following complete convergence result for weighted sums of NA random variables with identical distribution.

### Theorem 1.1

*Let* $\{X, X_{n}; n\geq1\}$ *be a sequence of NA random variables with identical distribution*, *and let* $\{ a_{ni},1\leq i\leq n, n\geq1\}$ *be a triangular array of constants satisfying* $\sum_{i=1}^{n} \vert a_{ni} \vert ^{\alpha}=O(n)$ *for some* $0<\alpha\leq2 $. *Let* $b_{n}=n^{1/\alpha}{(\log n)}^{1/\gamma }$ *for some* $\gamma>0$. *Furthermore*, *assume that* $EX=0$ *when* $1<\alpha \leq2 $. *If for some* $h>0$,

1.4 $$ E\exp \bigl(h \vert X \vert ^{\gamma} \bigr)< \infty, $$

*then*, *for all* $\varepsilon>0$,

1.5 $$ \sum_{n=1}^{\infty}\frac{1}{n} P \Biggl(\max_{1\leq j\leq n} \Biggl\vert \sum _{i=1}^{j} a_{ni} X_{i} \Biggr\vert >\varepsilon b_{n} \Biggr)< \infty. $$

Sung [2] extended the result of Cai [1] under a much weaker moment condition and obtained the following strong convergence results.

### Theorem 1.2

*Let* $\{X, X_{n}; n\geq1\}$ *be a sequence of NA random variables with identical distribution*, *and let* $\{a_{ni},1\leq i\leq n, n\geq1\}$ *be an array of constants such that* $\sum_{i=1}^{n} \vert a_{ni} \vert ^{\alpha}=O(n)$ *for some* $0<\alpha\leq2 $. *Let* $b_{n}=n^{1/\alpha}{(\log n)}^{1/\gamma}$ *for some* $\gamma>0$. *Furthermore*, *suppose that* $EX=0$ *when* $1<\alpha\leq2$. *Then*:

(i) *If* $\alpha>\gamma$, *then* $E \vert X \vert ^{\alpha }<\infty$ *implies* (1.5).
(ii) *If* $\alpha=\gamma$, *then* $E \vert X \vert ^{\alpha }\log(1+ \vert X \vert )<\infty$ *implies* (1.5).
(iii) *If* $\alpha<\gamma$, *then* $E \vert X \vert ^{\gamma }<\infty$ *implies* (1.5).

In the case $\alpha>\gamma$, Chen and Sung [3] studied the complete convergence for weighted sums of NA random variables under the moment condition $E \vert X \vert ^{\alpha}/ (\log(1+ \vert X \vert ) ) ^{\alpha/\gamma-1} <\infty$, which is weaker than Theorem 1.2. Li *et al.* [21] extended and improved the result of Chen and Sung [3] to $\rho^{*}$-mixing random variables. Motivated by the above results obtained by Cai [1], Sung [2] and Chen and Sung [3], in this paper, we will further study the complete convergence for weighted sums of NSD random variables. Some complete convergence results for the maximum weighted sums of NSD random variables are obtained without the assumption of an identical distribution. As an application, the Marcinkiewicz-Zygmund type strong law of large numbers for weighted sums of NSD random variables is obtained. Our results not only generalize the corresponding ones of Cai [1] and Sung [2], but they also extend and improve the corresponding one of Chen and Sung [3].

## Preliminaries

Throughout this paper, *C* represents a generic positive constant whose value may change from one appearance to the next, and $a_{n}=O(b_{n})$ means $a_{n}\leq Cb_{n}$. Let $I(A)$ be the indicator function of the set *A*.

### Definition 2.1

A sequence of random variables $\{X_{n};n\geq1\}$ is said to be stochastically dominated by a random variable *X* if there exists a positive constant *C* such that

$$\begin{aligned} P \bigl( \vert X_{n} \vert \geq x \bigr)\leq CP \bigl( \vert X \vert \geq x \bigr), \end{aligned} $$

for all $x\geq0$ and $n\geq1$.

In order to prove our main results, we introduce the following lemmas.

### Lemma 2.1

(Hu [5])

*If* $X= (X_{1}, X_{2},\ldots ,X_{n} )$ *is NSD and* $f_{1}, f_{2},\ldots,f_{n}$ *are nondecreasing functions*, *then* $(f_{1}(X_{1}), f_{2}(X_{2}),\ldots ,f_{n}(X_{n}) )$ *is NSD*.

### Lemma 2.2

(Wang *et al.* [11])

*Let* $p>1$ *and* $\{X_{n};n\geq1\}$ *be a sequence of NSD random variables with* $E \vert X_{i} \vert ^{p}<\infty$ *for every* $i\geq1$. *Then*, *there exists a positive constant* $C=C_{p}$ *depending only on* *p* *such that*, *for every* $n\geq1 $, *for* $1< p\leq2$,

$$\begin{aligned} E \Biggl(\max_{1\leq j\leq n} \Biggl\vert \sum_{i=1}^{j} X_{i} \Biggr\vert ^{p} \Biggr)\leq C\sum_{i=1}^{n} E \vert X_{i} \vert ^{p}, \end{aligned} $$

*and*, *for* $p>2$,

$$\begin{aligned} E \Biggl(\max_{1\leq j\leq n} \Biggl\vert \sum_{i=1}^{j} X_{i} \Biggr\vert ^{p} \Biggr)\leq C \Biggl\{ \sum_{i=1}^{n} E \vert X_{i} \vert ^{p}+ \Biggl(\sum _{i=1}^{n} EX_{i}^{2} \Biggr)^{p/2} \Biggr\} . \end{aligned} $$

### Lemma 2.3

(Sung [22])

*Let* *X* *be a random variable and* $\{a_{ni};1\leq i\leq n,n\geq1\}$ *be an array of constants satisfying* $\sum_{i=1}^{n} \vert a_{ni} \vert ^{\alpha}=O (n )$ *for some* $\alpha> 0$. *Let* $b_{n}=n^{1/\alpha }{(\log n)}^{1/\gamma}$ *for some* $\gamma> 0$. *Then*

2.1 $$ \sum_{n=1}^{\infty}\frac{1}{n}\sum _{i=1}^{n} P \bigl( \vert a_{ni}X \vert >b_{n} \bigr)\leq \textstyle\begin{cases} {CE \vert X \vert ^{\alpha}}, &\textit{for }\alpha>\gamma, \\ {CE \vert X \vert ^{\alpha}\log(1+ \vert X \vert )}, &\textit{for } \alpha=\gamma, \\ {CE \vert X \vert ^{\gamma}}, &\textit{for }\alpha< \gamma. \end{cases} $$

### Lemma 2.4

(Sung [22])

*Let* *X* *be a random variable and* $\{a_{ni};1\leq i\leq n,n\geq1\}$ *be an array of constants satisfying* $a_{ni}=0$ *or* $\vert a_{ni} \vert >1$, *and* $\sum_{i=1}^{n} \vert a_{ni} \vert ^{\alpha}=O (n )$ *for some* $\alpha> 0$. *Let* $b_{n}=n^{1/\alpha}{(\log n)}^{1/\alpha}$. *If* $p>\alpha$, *then*

2.2 $$ \sum_{n=1}^{\infty}n^{-1}b_{n}^{-p}\sum_{i=1}^{n} E \vert a_{ni}X \vert ^{p}I \bigl( \vert a_{ni}X \vert \leq b_{n} \bigr)\leq CE \vert X \vert ^{\alpha}\log \bigl(1+ \vert X \vert \bigr). $$

### Lemma 2.5

(Wu [23])

*Let* $\{X_{n};n\geq1\}$ *be a sequence of random variables which is stochastically dominated by a random variable* *X*. *For any* $u>0$, $t>0$ *and* $n\geq1$, *the following two statements hold*:

2.3 $$\begin{aligned}& E \vert X_{n} \vert ^{u} I \bigl( \vert X_{n} \vert \leq t \bigr)\leq C \bigl[E \vert X \vert ^{u} I \bigl( \vert X \vert \leq t \bigr)+t^{u} P \bigl( \vert X \vert >t \bigr) \bigr], \end{aligned}$$

2.4 $$\begin{aligned}& E \vert X_{n} \vert ^{u} I \bigl( \vert X_{n} \vert > t \bigr)\leq CE \vert X \vert ^{u} I \bigl( \vert X \vert > t \bigr). \end{aligned}$$

## Main results and proofs

Now we state and prove our main results.

### Theorem 3.1

*Let* $\{X_{n};n\geq1\}$ *be a sequence of NSD random variables which is stochastically dominated by a random variable* *X*, *and* $b_{n}=n^{1/\alpha}{(\log n)}^{1/\gamma}$ *for some* $0<\alpha\leq2$ *and* $\gamma>0$. *Let* $\{a_{ni};1\leq i\leq n,n\geq1\}$ *be an array of constants satisfying* $\sum_{i=1}^{n} \vert a_{ni} \vert ^{\gamma}=O(n)$. *Assume further that* $EX_{n}=0$ *when* $1<\alpha \leq2$. *Then*:

(i) *If* $\alpha<\gamma$, *then* $E \vert X \vert ^{\gamma }<\infty$ *implies* (1.5).
(ii) *If* $\alpha=\gamma$, *then* $E \vert X \vert ^{\alpha }\log(1+ \vert X \vert )<\infty$ *implies* (1.5).

### Theorem 3.2

*Let* $\{X_{n};n\geq1\}$ *be a sequence of NSD random variables which is stochastically dominated by a random variable* *X*, *and* $b_{n}=n^{1/\alpha}{(\log n)}^{1/\gamma}$ *for some* $0<\alpha\leq2$ *and* $\gamma>0$. *Let* $\{a_{ni};1\leq i\leq n,n\geq1\}$ *be an array of constants satisfying* $\sum_{i=1}^{n} \vert a_{ni} \vert ^{\alpha}=O(n)$. *Assume further that* $EX_{n}=0$ *when* $1<\alpha \leq2$. *If* $\alpha>\gamma$, *then* $E \vert X \vert ^{\alpha }/ (\log(1+ \vert X \vert ) )^{\alpha/\gamma-1}<\infty $ *implies* (1.5).

### Remark 3.1

In Theorem 3.1 and Theorem 3.2, we use different methods from those of Sung [2] and Chen and Sung [3] to prove the results, and obtain some strong convergence results for weighted sums of NSD random variables without assumptions of identical distribution. The obtained theorems not only extend the corresponding results of Cai [1] and Sung [2] and Chen and Sung [3] to the case of NSD random variables, but they also improve them.

### Proof of Theorem 3.1

Without loss of generality, we suppose that $a_{ni}>0$. For $\forall i\geq1$, define

$$\begin{gathered} Y_{i}=-b_{n}I (a_{ni}X_{ni}< -b_{n} )+a_{ni}X_{ni}I \bigl( \vert a_{ni}X_{ni} \vert \leq b_{n} \bigr)+b_{n}I (a_{ni}X_{ni}>b_{n} ), \\ T_{j}=\sum_{i=1}^{j} (Y_{i}-EY_{i} ),\quad j=1,2,\ldots,n. \end{gathered} $$

It is easy to check that, for all $\varepsilon>0$,

3.1 $$ \Biggl\{ \max_{1\leq j\leq n} \Biggl\vert \sum _{i=1}^{j} a_{ni}X_{i} \Biggr\vert >\varepsilon b_{n} \Biggr\} \subset \Bigl\{ \max _{1\leq j\leq n} \vert a_{nj}X_{j} \vert >b_{n} \Bigr\} \cup \Biggl\{ \max_{1\leq j\leq n} \Biggl\vert \sum_{i=1}^{j} Y_{i} \Biggr\vert >\varepsilon b_{n} \Biggr\} , $$

which implies that

3.2 $$\begin{aligned} &P \Biggl(\max_{1\leq j\leq n} \Biggl\vert \sum _{i=1}^{j} a_{ni}X_{i} \Biggr\vert >\varepsilon b_{n} \Biggr) \\ &\quad \leq P \Biggl(\max_{1\leq j\leq n} \Biggl\vert \sum _{i=1}^{j} Y_{i} \Biggr\vert >\varepsilon b_{n} \Biggr)+P \Bigl(\max_{1\leq j\leq n} \vert a_{nj}X_{j} \vert >b_{n} \Bigr) \\ &\quad \leq P \Biggl(\max_{1\leq j\leq n} \vert T_{j} \vert > \varepsilon b_{n}-\max_{1\leq j\leq n} \Biggl\vert \sum _{i=1}^{j} EY_{i} \Biggr\vert \Biggr)+\sum_{j=1}^{n} P \bigl( \vert a_{nj}X_{j} \vert >b_{n} \bigr). \end{aligned}$$

Firstly, we prove that

3.3 $$ b_{n}^{-1}\max_{1\leq j\leq n} \Biggl\vert \sum_{i=1}^{j} EY_{i} \Biggr\vert \rightarrow0,\quad \text{as }n \to\infty. $$

If $1<\alpha\leq2$, then by $EX_{n}=0$, Lemma 2.5, Definition 2.1, the $C_{r}$ inequality, the Markov inequality and the Hölder inequality, we get

3.4 $$\begin{aligned} b_{n}^{-1}\max_{1\leq j\leq n} \Biggl\vert \sum _{i=1}^{j}EY_{i} \Biggr\vert \leq& b_{n}^{-1}\sum_{i=1}^{n} \vert EY_{i} \vert \\ \leq& b_{n}^{-1}\sum_{i=1}^{n} \bigl\vert Ea_{ni}X_{i}I \bigl( \vert a_{ni}X_{i} \vert \leq b_{n} \bigr) \bigr\vert +\sum_{i=1}^{n}P \bigl( \vert a_{ni}X_{i} \vert > b_{n} \bigr) \\ \leq& Cb_{n}^{-1}\sum_{i=1}^{n}E \vert a_{ni}X \vert I \bigl( \vert a_{ni}X \vert > b_{n} \bigr) +C\sum_{i=1}^{n}P \bigl( \vert a_{ni}X \vert > b_{n} \bigr) \\ \leq& Cb_{n}^{-\alpha}\sum_{i=1}^{n}E \vert a_{ni}X \vert ^{\alpha }I \bigl( \vert a_{ni}X \vert > b_{n} \bigr) +Cb_{n}^{-\alpha} \sum_{i=1}^{n}E \vert a_{ni}X \vert ^{\alpha } \\ \leq& Cb_{n}^{-\alpha}\sum_{i=1}^{n}E \vert a_{ni}X \vert ^{\alpha} +Cb_{n}^{-\alpha} \sum_{i=1}^{n}E \vert a_{ni}X \vert ^{\alpha } \\ \leq& Cb_{n}^{-\alpha}\sum_{i=1}^{n} \vert a_{ni} \vert ^{\gamma }E \vert X \vert ^{\alpha} \\ \leq& C (\log n )^{-\alpha/\gamma}\rightarrow0,\quad \text{as } n\rightarrow \infty. \end{aligned}$$

If $0<\alpha\leq1$, then by Lemma 2.5, Definition 2.1, the $C_{r}$ inequality and the Markov inequality, we get again

3.5 $$\begin{aligned} b_{n}^{-1}\max_{1\leq j\leq n} \Biggl\vert \sum _{i=1}^{j}EY_{i} \Biggr\vert \leq& b_{n}^{-1}\sum_{i=1}^{n} \vert EY_{i} \vert \\ \leq& b_{n}^{-1}\sum_{i=1}^{n} \bigl\vert Ea_{ni}X_{i}I \bigl( \vert a_{ni}X_{i} \vert \leq b_{n} \bigr) \bigr\vert +\sum_{i=1}^{n}P \bigl( \vert a_{ni}X_{i} \vert > b_{n} \bigr) \\ \leq& Cb_{n}^{-1}\sum_{i=1}^{n} \bigl[E \vert a_{ni}X \vert I \bigl( \vert a_{ni}X \vert \leq b_{n} \bigr)+b_{n}P \bigl( \vert a_{ni}X \vert > b_{n} \bigr) \bigr] \\ &{}+C\sum_{i=1}^{n}P \bigl( \vert a_{ni}X \vert > b_{n} \bigr) \\ \leq& Cb_{n}^{-\alpha}\sum_{i=1}^{n}E \vert a_{ni}X \vert ^{\alpha} +Cb_{n}^{-\alpha} \sum_{i=1}^{n}E \vert a_{ni}X \vert ^{\alpha } \\ \leq& Cb_{n}^{-\alpha}\sum_{i=1}^{n} \vert a_{ni} \vert ^{\gamma }E \vert X \vert ^{\alpha} \\ \leq& C (\log n )^{-\alpha/\gamma}\rightarrow0,\quad \text{as } n\rightarrow \infty. \end{aligned}$$

It immediately follows from (3.4) and (3.5), that (3.3) holds. Hence, for *n* large enough,

3.6 $$ P \Biggl(\max_{1\leq j\leq n} \Biggl\vert \sum _{i=1}^{j}a_{ni}X_{i} \Biggr\vert >\varepsilon b_{n} \Biggr)\leq\sum_{i=1}^{n}P \bigl( \vert a_{ni}X_{i} \vert > b_{n} \bigr)+P \biggl(\max_{1\leq j\leq n} \biggl\vert T_{j}> \frac{\varepsilon b_{n}}{2} \biggr\vert \biggr). $$

Then, to prove (1.5), it suffices to prove that

3.7 $$ I\triangleq\sum_{n=1}^{\infty} \frac{1}{n}\sum_{i=1}^{n}P \bigl( \vert a_{ni}X_{i} \vert >b_{n} \bigr)< \infty $$

and

3.8 $$ J\triangleq\sum_{n=1}^{\infty} \frac{1}{n}P \biggl(\max_{1\leq j\leq n} \vert T_{j} \vert >\frac{\varepsilon b_{n}}{2} \biggr)< \infty. $$

By Lemma 2.3, we can easily obtain

3.9 $$\begin{aligned} I \triangleq& \sum_{n=1}^{\infty} \frac{1}{n}\sum_{i=1}^{n}P \bigl( \vert a_{ni}X_{i} \vert >b_{n} \bigr) \\ \leq& C\sum_{n=1}^{\infty}\frac{1}{n} \sum_{i=1}^{n}P \bigl( \vert a_{ni}X \vert >b_{n} \bigr)< \infty. \end{aligned}$$

For fixed $n\geq1$, it is easily seen that $\{Y_{i};1\leq i\leq n\}$ is still a sequence of NSD random variables by Lemma 2.1. Hence, for $p>2$, it follows from Lemma 2.2, the Markov inequality and the Jensen inequality that

3.10 $$\begin{aligned} J \triangleq& \sum_{n=1}^{\infty} \frac{1}{n}P \biggl(\max_{1\leq j\leq n} \vert T_{j} \vert >\frac{\varepsilon b_{n}}{2} \biggr) \\ \leq& C\sum_{n=1}^{\infty} \frac{1}{n}b_{n}^{-p}E \Bigl(\max_{1\leq j\leq n} \vert T_{j} \vert ^{p} \Bigr) \\ \leq& C\sum_{n=1}^{\infty}n^{-1}b_{n}^{-p} \Biggl(\sum_{i=1}^{n}E \vert Y_{i}-EY_{i} \vert ^{p} + \Biggl(\sum _{i=1}^{n}E \vert Y_{i}-EY_{i} \vert ^{2} \Biggr)^{p/2} \Biggr) \\ \leq& C\sum_{n=1}^{\infty}n^{-1}b_{n}^{-p} \sum_{i=1}^{n}E \vert Y_{i} \vert ^{p} +C\sum_{n=1}^{\infty}n^{-1}b_{n}^{-p} \Biggl(\sum_{i=1}^{n}E \vert Y_{i} \vert ^{2} \Biggr)^{p/2} \\ \triangleq& J_{1}+J_{2}. \end{aligned}$$

Firstly, we prove $J_{1}<\infty$. By Lemma 2.3, we obtain

3.11 $$\begin{aligned} J_{1} \triangleq& C\sum_{n=1}^{\infty}n^{-1}b_{n}^{-p} \sum_{i=1}^{n}E \vert Y_{i} \vert ^{p} \\ \leq& C\sum_{n=1}^{\infty}n^{-1}b_{n}^{-p} \sum_{i=1}^{n} \bigl[E \vert a_{ni}X \vert ^{p} I \bigl( \vert a_{ni}X \vert \leq b_{n} \bigr)+b_{n}^{p}P \bigl( \vert a_{ni}X \vert > b_{n} \bigr) \bigr] \\ =&C\sum_{n=1}^{\infty}n^{-1}b_{n}^{-p} \sum_{i=1}^{n}E \vert a_{ni}X \vert ^{p} I \bigl( \vert a_{ni}X \vert \leq b_{n} \bigr) +C\sum_{n=1}^{\infty}n^{-1} \sum_{i=1}^{n}P \bigl( \vert a_{ni}X \vert > b_{n} \bigr) \\ \triangleq& J_{11}+J_{12}. \end{aligned}$$

Actually, by Lemma 2.3, we can directly obtain $J_{12}<\infty$. Hence, we only need to prove $J_{11}<\infty$ in the following two cases.

(i) If $\alpha<\gamma$, take $p>\max \{2,\gamma \}$, then by $\sum_{i=1}^{n} \vert a_{ni} \vert ^{\gamma}\leq Cn$ and $E \vert X \vert ^{\gamma}<\infty$ it follows that

3.12 $$\begin{aligned} J_{11} &=C\sum_{n=1}^{\infty}n^{-1}b_{n}^{-p} \sum_{i=1}^{n}E \vert a_{ni}X \vert ^{p} I \bigl( \vert a_{ni}X \vert \leq b_{n} \bigr) \\ &=C\sum_{n=1}^{\infty}n^{-1}\sum _{i=1}^{n}E \biggl\vert \frac {a_{ni}X}{b_{n}} \biggr\vert ^{p} I \bigl( \vert a_{ni}X \vert \leq b_{n} \bigr) \\ &\leq C\sum_{n=1}^{\infty}n^{-1}\sum _{i=1}^{n}E \biggl\vert \frac {a_{ni}X}{b_{n}} \biggr\vert ^{\gamma} I \bigl( \vert a_{ni}X \vert \leq b_{n} \bigr) \\ &\leq C\sum_{n=1}^{\infty}n^{-1}b_{n}^{-\gamma} \sum_{i=1}^{n}E \vert a_{ni}X \vert ^{\gamma} I \bigl( \vert a_{ni}X \vert \leq b_{n} \bigr) \\ &\leq C\sum_{n=1}^{\infty}n^{-1}b_{n}^{-\gamma} \sum_{i=1}^{n} \vert a_{ni} \vert ^{\gamma} E \vert X \vert ^{\gamma} \\ & \begin{aligned}[b] &\leq C\sum_{n=1}^{\infty}n^{-1}n^{-\gamma/\alpha} (\log{n} )^{-1}nE \vert X \vert ^{\gamma} \\ &\leq C\sum_{n=1}^{\infty}n^{-\gamma/\alpha} ( \log{n} )^{-1}< \infty. \end{aligned} \end{aligned}$$

(ii) If $\alpha=\gamma$, we need to divide $\{a_{ni};1\leq i\leq n,n\geq1 \}$ into three subsets: $\{a_{ni}: \vert a_{ni} \vert \leq1/ (\log{n} )^{t} \}$, $\{a_{ni}:1/ (\log{n} )^{t}< \vert a_{ni} \vert \leq1 \}$ and $\{a_{ni}: \vert a_{ni} \vert >1 \}$, where $t=1/(p-{\alpha})$. Then we obtain

3.13 $$\begin{aligned} J_{11} =&C\sum_{n=1}^{\infty}n^{-1}b_{n}^{-p} \sum_{i=1}^{n}E \vert a_{ni}X \vert ^{p} I \bigl( \vert a_{ni}X \vert \leq b_{n} \bigr) \\ =&C\sum_{n=1}^{\infty}n^{-1}b_{n}^{-p} \sum_{i: \vert a_{ni} \vert \leq1/ (\log{n} )^{t}}^{n}E \vert a_{ni}X \vert ^{p}I \bigl( \vert a_{ni}X \vert \leq b_{n} \bigr) \\ &{}+C\sum_{n=1}^{\infty}n^{-1}b_{n}^{-p} \sum_{i:1/ (\log{n} )^{t}< \vert a_{ni} \vert \leq1}^{n}E \vert a_{ni}X \vert ^{p}I \bigl( \vert a_{ni}X \vert \leq b_{n} \bigr) \\ &{}+C\sum_{n=1}^{\infty}n^{-1}b_{n}^{-p} \sum_{i: \vert a_{ni} \vert >1}^{n}E \vert a_{ni}X \vert ^{p}I \bigl( \vert a_{ni}X \vert \leq b_{n} \bigr) \\ \triangleq& J_{111}+J_{112}+J_{113}. \end{aligned}$$

Obviously, by Lemma 2.4, we directly obtain $J_{113}\leq E \vert X \vert ^{\alpha}\log{(1+{ \vert X \vert )}}<\infty$.

It follows from $\sum_{i: \vert a_{ni} \vert \leq1/ (\log {n} )^{t}} \vert a_{ni} \vert ^{\alpha} \leq Cn (\log{n} )^{-t\alpha}$ and $E \vert X \vert ^{\alpha}<\infty$ that

3.14 $$\begin{aligned} J_{111} =& C\sum_{n=1}^{\infty}n^{-1}b_{n}^{-p} \sum_{i: \vert a_{ni} \vert \leq1/ (\log{n} )^{t}}^{n}E \vert a_{ni}X \vert ^{p}I \bigl( \vert a_{ni}X \vert \leq b_{n} \bigr) \\ \leq& C\sum_{n=1}^{\infty}n^{-1}b_{n}^{-p} \sum_{i: \vert a_{ni} \vert \leq1/ (\log{n} )^{t}}^{n} \vert a_{ni} \vert ^{p} E \vert X \vert ^{p}I \bigl( \vert a_{ni}X \vert \leq b_{n} \bigr) \\ \leq& C\sum_{n=1}^{\infty}n^{-1}b_{n}^{-\alpha} \sum_{i: \vert a_{ni} \vert \leq1/ (\log{n} )^{t}}^{n} \vert a_{ni} \vert ^{\alpha} E \vert X \vert ^{\alpha}I \bigl( \vert a_{ni}X \vert \leq b_{n} \bigr) \\ \leq& C\sum_{n=1}^{\infty}n^{-1}b_{n}^{-\alpha}E \vert X \vert ^{\alpha}\sum_{i: \vert a_{ni} \vert \leq1/ (\log {n} )^{t}}^{n} \vert a_{ni} \vert ^{\alpha} \\ \leq& C\sum_{n=1}^{\infty}n^{-1}b_{n}^{-\alpha}E \vert X \vert ^{\alpha}\sum_{i: \vert a_{ni} \vert \leq1/ (\log {n} )^{t}}^{n} \vert a_{ni} \vert ^{\alpha} \\ \leq& C\sum_{n=1}^{\infty}E \vert X \vert ^{\alpha}n^{-1} (\log{n})^{-1-t{\alpha}} \\ \leq& C\sum_{n=1}^{\infty}n^{-1}( \log{n})^{-1-t{\alpha}}< \infty. \end{aligned}$$

It follows from $\sum_{i:1/ (\log{n} )^{t}< \vert a_{ni} \vert \leq1} \vert a_{ni} \vert ^{p}\leq Cn$, $E \vert X \vert ^{\alpha}<\infty$ and $t=1/(p-{\alpha})$ for $p>2$, $0<\alpha\leq2$ that

3.15 $$\begin{aligned} J_{112} =&C\sum_{n=1}^{\infty}n^{-1}b_{n}^{-p} \sum_{i:1/ (\log{n} )^{t}< \vert a_{ni} \vert \leq1}^{n}E \vert a_{ni}X \vert ^{p}I \bigl( \vert a_{ni}X \vert \leq b_{n} \bigr) \\ \leq& C\sum_{n=1}^{\infty}n^{-1}b_{n}^{-p} \sum_{i:1/ (\log{n} )^{t}< \vert a_{ni} \vert \leq1}^{n} \vert a_{ni} \vert ^{p} E \vert X \vert ^{p}I \bigl( \vert a_{ni}X \vert \leq b_{n} \bigr) \\ \leq& C\sum_{n=1}^{\infty}b_{n}^{-p}E \vert X \vert ^{p} I \bigl( \vert X \vert \leq b_{n} (\log{n} )^{t} \bigr) \\ \leq& C\sum_{n=1}^{\infty}b_{n}^{-p} \sum_{k=1}^{n}E \vert X \vert ^{p} I \bigl( (k-1 )^{1/\alpha} \bigl(\log{ (k-1 )} \bigr)^{t+1/{\alpha}} < \vert X \vert \leq k^{1/\alpha} (\log{k} )^{t+1/{\alpha}} \bigr) \\ \leq& C\sum_{k=1}^{\infty}E \vert X \vert ^{p}I \bigl( (k-1 )^{1/\alpha} \bigl(\log{ (k-1 )} \bigr)^{t+1/{\alpha}} < \vert X \vert \leq k^{1/\alpha} (\log{k} )^{t+1/{\alpha}} \bigr) \\ &{}\times\sum_{n=k}^{\infty}n^{-p/{\alpha}} ( \log{n} )^{-p/{\alpha}} \\ \leq& C\sum_{k=1}^{\infty}E \vert X \vert ^{\alpha}\frac{k^{p/{\alpha}} (\log{k} )^{p (t+1/{\alpha} )}}{ (k-1 ) (\log{ (k-1 )} )^{{\alpha}t+1}}k^{1-p/{\alpha}} (\log{k} )^{-p/{\alpha }} \\ \leq& CE \vert X \vert ^{\alpha}< \infty. \end{aligned}$$

Therefore, by (3.11)-(3.15), we can see that $J_{1}<\infty$. Finally, we prove $J_{2}<\infty$. Actually, take $p>\max \{2,\frac {2\gamma}{\alpha} \}$, then by Lemma 2.5, the Markov inequality and $E \vert X \vert ^{\gamma}<\infty$, we get

3.16 $$\begin{aligned} J_{2} =& C\sum_{n=1}^{\infty}n^{-1}b_{n}^{-p} \Biggl(\sum_{i=1}^{n}E \vert Y_{i} \vert ^{2} \Biggr)^{p/2} \\ \leq& C\sum_{n=1}^{\infty}n^{-1}b_{n}^{-p} \Biggl(\sum_{i=1}^{n}E \vert a_{ni}X_{i} \vert ^{2}I \bigl( \vert a_{ni}X_{i} \vert \leq b_{n} \bigr) \Biggr)^{p/2} \\ &{}+C\sum_{n=1}^{\infty}n^{-1}b_{n}^{-p} \Biggl(\sum_{i=1}^{n}b_{n}^{2} P \bigl( \vert a_{ni}X_{i} \vert > b_{n} \bigr) \Biggr)^{p/2} \\ \leq& C\sum_{n=1}^{\infty}n^{-1}b_{n}^{-p} \Biggl(\sum_{i=1}^{n} \bigl[E \vert a_{ni}X \vert ^{2}I \bigl( \vert a_{ni}X \vert \leq b_{n} \bigr) +b_{n}^{2}P \bigl( \vert a_{ni}X \vert > b_{n} \bigr) \bigr] \Biggr)^{p/2} \\ &{}+C\sum_{n=1}^{\infty}n^{-1} \Biggl( \sum_{i=1}^{n} P \bigl( \vert a_{ni}X \vert > b_{n} \bigr) \Biggr)^{p/2} \\ \leq& C\sum_{n=1}^{\infty}n^{-1} \Biggl(\sum_{i=1}^{n}b_{n}^{-2}E \vert a_{ni}X \vert ^{2}I \bigl( \vert a_{ni}X \vert \leq b_{n} \bigr) \Biggr)^{p/2} \\ &{}+C\sum_{n=1}^{\infty}n^{-1} \Biggl( \sum_{i=1}^{n} P \bigl( \vert a_{ni}X \vert > b_{n} \bigr) \Biggr)^{p/2} \\ \leq& C\sum_{n=1}^{\infty}n^{-1} \Biggl(\sum_{i=1}^{n}b_{n}^{-\alpha}E \vert a_{ni}X \vert ^{\alpha}I \bigl( \vert a_{ni}X \vert \leq b_{n} \bigr) \Biggr)^{p/2} \\ &{}+C\sum_{n=1}^{\infty}n^{-1} \Biggl( \sum_{i=1}^{n}b_{n}^{-\alpha}E \vert a_{ni}X \vert ^{\alpha} \Biggr)^{p/2} \\ \leq& C\sum_{n=1}^{\infty}n^{-1}b_{n}^{{-\alpha}p/2} \Biggl(\sum_{i=1}^{n} \vert a_{ni} \vert ^{\alpha}E \vert X \vert ^{\alpha} \Biggr)^{p/2} \\ \leq& C\sum_{n=1}^{\infty}n^{-1}b_{n}^{{-\alpha}p/2}n^{p/2} \\ =&C\sum_{n=1}^{\infty}n^{-1}n^{-p/2} (\log{n} )^{-\frac {\alpha p}{2\gamma}}n^{p/2} \\ =&C\sum_{n=1}^{\infty}n^{-1} ( \log{n} )^{-\frac{\alpha p}{2\gamma}}< \infty. \end{aligned}$$

Thus, the proof of Theorem 3.1 is completed. □

### Proof of Theorem 3.2

Without loss of generality, we suppose that $a_{ni}>0$. For $\forall i\geq1$, define

$$\begin{aligned} Z_{i}=a_{ni}X_{ni}I \bigl( \vert a_{ni}X_{ni} \vert \leq b_{n} \bigr). \end{aligned} $$

It is easy to check that, for all $\varepsilon>0$,

$$\begin{aligned} \Biggl\{ \max_{1\leq j\leq n} \Biggl\vert \sum_{i=1}^{j} a_{ni}X_{i} \Biggr\vert >\varepsilon b_{n} \Biggr\} \subset \Bigl\{ \max _{1\leq j\leq n} \vert a_{nj}X_{j} \vert >b_{n} \Bigr\} \cup \Biggl\{ \max_{1\leq j\leq n} \Biggl\vert \sum_{i=1}^{j} Z_{i} \Biggr\vert >\varepsilon b_{n} \Biggr\} , \end{aligned} $$

which implies that

3.17 $$\begin{aligned} &P \Biggl(\max_{1\leq j\leq n} \Biggl\vert \sum _{i=1}^{j} a_{ni}X_{i} \Biggr\vert >\varepsilon b_{n} \Biggr) \\ &\quad \leq P \Biggl(\max_{1\leq j\leq n} \Biggl\vert \sum _{i=1}^{j} Z_{i} \Biggr\vert >\varepsilon b_{n} \Biggr)+P \Bigl(\max_{1\leq j\leq n} \vert a_{nj}X_{j} \vert >b_{n} \Bigr) \\ &\quad \leq P \Biggl(\max_{1\leq j\leq n} \Biggl\vert \sum _{i=1}^{j} Z_{i} \Biggr\vert >\varepsilon b_{n} \Biggr)+\sum_{j=1}^{n} P \bigl( \vert a_{nj}X_{j} \vert >b_{n} \bigr). \end{aligned}$$

To prove (1.5), it suffices to show that

3.18 $$ H\triangleq\sum_{n=1}^{\infty} \frac{1}{n}\sum_{i=1}^{n}P \bigl( \vert a_{ni}X_{i} \vert >b_{n} \bigr)< \infty $$

and

3.19 $$ L\triangleq\sum_{n=1}^{\infty} \frac{1}{n}P \Biggl(\max_{1\leq j\leq n} \Biggl\vert \sum _{i=1}^{j} Z_{i} \Biggr\vert >\varepsilon b_{n} \Biggr)< \infty. $$

We first prove (3.18). Note that

3.20 $$ P \bigl( \vert a_{ni}X_{i} \vert >b_{n} \bigr)=P \bigl( \vert a_{ni}X_{i} \vert >b_{n}, \vert X_{i} \vert >b_{n} \bigr) +P \bigl( \vert a_{ni}X_{i} \vert >b_{n}, \vert X_{i} \vert \leq b_{n} \bigr). $$

By the Markov inequality, we get

3.21 $$ P \bigl( \vert a_{ni}X_{i} \vert >b_{n}, \vert X_{i} \vert >b_{n} \bigr) \leq b_{n}^{-\theta} \vert a_{ni} \vert ^{\theta}E \vert X_{i} \vert ^{\theta}I \bigl( \vert X_{i} \vert >b_{n} \bigr) $$

for any $0<\theta<\alpha$ and

3.22 $$ P \bigl( \vert a_{ni}X_{i} \vert >b_{n}, \vert X_{i} \vert \leq b_{n} \bigr) \leq b_{n}^{-\alpha} \vert a_{ni} \vert ^{\alpha}E \vert X_{i} \vert ^{\alpha}I \bigl( \vert X_{i} \vert \leq b_{n} \bigr). $$

It is easy to show that

3.23 $$\begin{aligned} &\sum_{n=1}^{\infty}n^{-1}b_{n}^{-\theta} \sum_{i=1}^{n} \vert a_{ni} \vert ^{\theta} E \vert X_{i} \vert ^{\theta}I \bigl( \vert X_{i} \vert >b_{n} \bigr) \\ &\quad \leq C\sum_{n=1}^{\infty}b_{n}^{-\theta}E \vert X \vert ^{\theta}I \bigl( \vert X \vert >b_{n} \bigr) \\ &\quad \leq CE \vert X \vert ^{\alpha}/ \bigl(\log \bigl(1+ \vert X \vert \bigr) \bigr)^{\alpha/\gamma }< \infty \end{aligned}$$

and

3.24 $$\begin{aligned} &\sum_{n=1}^{\infty}n^{-1}b_{n}^{-\alpha} \sum_{i=1}^{n} \vert a_{ni} \vert ^{\alpha} E \vert X_{i} \vert ^{\alpha}I \bigl( \vert X_{i} \vert \leq b_{n} \bigr) \\ &\quad \leq C\sum_{n=1}^{\infty}b_{n}^{-\alpha} \bigl[E \vert X \vert ^{\alpha}I \bigl( \vert X \vert \leq b_{n} \bigr) +b_{n}^{\alpha}P \bigl( \vert X \vert >b_{n} \bigr) \bigr] \\ &\quad \leq C\sum_{n=1}^{\infty}b_{n}^{-\alpha}E \vert X \vert ^{\alpha}I \bigl( \vert X \vert \leq b_{n} \bigr) +C\sum_{n=1}^{\infty} P \bigl( \vert X \vert >n^{1/\alpha} (\log{n} )^{1/\gamma} \bigr) \\ &\quad \leq CE \vert X \vert ^{\alpha}/ \bigl(\log \bigl(1+ \vert X \vert \bigr) \bigr)^{\alpha/\gamma-1} +CE \vert X \vert ^{\alpha}/ \bigl( \log \bigl(1+ \vert X \vert \bigr) \bigr)^{\alpha/\gamma }< \infty. \end{aligned}$$

Then, (3.18) holds by (3.20)-(3.24). Now we prove (3.19) in the following two cases.

(i) If $0<\alpha\leq1$, similar to the proof of (3.18), we have

3.25 $$\begin{aligned} E \vert a_{ni}X_{i} \vert ^{\alpha}I \bigl( \vert a_{ni}X_{i} \vert \leq b_{n} \bigr) =&E \vert a_{ni}X_{i} \vert ^{\alpha}I \bigl( \vert a_{ni}X_{i} \vert \leq b_{n}, \vert X_{i} \vert >b_{n} \bigr) \\ &{}+E \vert a_{ni}X_{i} \vert ^{\alpha}I \bigl( \vert a_{ni}X_{i} \vert \leq b_{n}, \vert X_{i} \vert \leq b_{n} \bigr). \end{aligned}$$

Note that

3.26 $$ E \vert a_{ni}X_{i} \vert ^{\alpha}I \bigl( \vert a_{ni}X_{i} \vert \leq b_{n}, \vert X_{i} \vert >b_{n} \bigr) \leq b_{n}^{\alpha-\theta} \vert a_{ni} \vert ^{\theta}E \vert X_{i} \vert ^{\theta}I \bigl( \vert X_{i} \vert >b_{n} \bigr) $$

for any $0<\theta<\alpha$ and

3.27 $$ E \vert a_{ni}X_{i} \vert ^{\alpha}I \bigl( \vert a_{ni}X_{i} \vert \leq b_{n}, \vert X_{i} \vert \leq b_{n} \bigr) \leq \vert a_{ni} \vert ^{\alpha}E \vert X_{i} \vert ^{\alpha}I \bigl( \vert X_{i} \vert \leq b_{n} \bigr). $$

By the Markov inequality, the $C_{r}$ inequality and (3.23)-(3.27), we obtain

3.28 $$ L\leq\sum_{n=1}^{\infty}n^{-1}b_{n}^{-\alpha} \sum_{i=1}^{n}E \vert a_{ni}X_{i} \vert ^{\alpha} I \bigl( \vert a_{ni}X_{i} \vert \leq b_{n} \bigr)< \infty. $$

(ii) If $1<\alpha\leq2$, we first prove that

3.29 $$ b_{n}^{-1}\max_{1\leq j\leq n} \Biggl\vert \sum _{i=1}^{j}Ea_{ni}X_{i} I \bigl( \vert a_{ni}X_{i} \vert \leq b_{n} \bigr) \Biggr\vert \rightarrow0,\quad\text{as } n \to\infty. $$

By $EX_{n}=0$, we have

3.30 $$\begin{aligned} \max_{1\leq j\leq n} \Biggl\vert \sum_{i=1}^{j}Ea_{ni}X_{i} I \bigl( \vert a_{ni}X_{i} \vert \leq b_{n} \bigr) \Biggr\vert \leq& \sum_{i=1}^{n}E \vert a_{ni}X_{i} \vert I \bigl( \vert a_{ni}X_{i} \vert >b_{n} \bigr) \\ =&\sum_{i=1}^{n}E \vert a_{ni}X_{i} \vert I \bigl( \vert a_{ni}X_{i} \vert >b_{n}, \vert X_{i} \vert >b_{n} \bigr) \\ &{}+\sum_{i=1}^{n}E \vert a_{ni}X_{i} \vert I \bigl( \vert a_{ni}X_{i} \vert >b_{n}, \vert X_{i} \vert \leq b_{n} \bigr). \end{aligned}$$

Since

3.31 $$\begin{aligned} &E \vert a_{ni}X_{i} \vert I \bigl( \vert a_{ni}X_{i} \vert >b_{n}, \vert X_{i} \vert >b_{n} \bigr) \\ &\quad \leq \vert a_{ni} \vert E \vert X \vert I \bigl( \vert X \vert >b_{n} \bigr) \\ &\quad = \vert a_{ni} \vert E \biggl(\frac{ \vert X \vert ^{\alpha}}{ (\log(1+ \vert X \vert ) )^{\alpha/\gamma-1}} \cdot \vert X \vert ^{1-\alpha} \bigl(\log\bigl(1+ \vert X \vert \bigr) \bigr)^{\alpha/\gamma-1} \biggr) I \bigl( \vert X \vert >b_{n} \bigr) \\ &\quad \leq Cb_{n}^{1-\alpha} \bigl(\log{(1+b_{n})} \bigr)^{\alpha/\gamma -1} \vert a_{ni} \vert \\ &\quad \leq Cn^{-1+1/\alpha} (\log{n} )^{1/\gamma-1} \vert a_{ni} \vert \end{aligned}$$

and

3.32 $$\begin{aligned} &E \vert a_{ni}X_{i} \vert I \bigl( \vert a_{ni}X_{i} \vert >b_{n}, \vert X_{i} \vert \leq b_{n} \bigr) \\ &\quad \leq E \vert a_{ni}X_{i} \vert \cdot \frac{ \vert a_{ni}X_{i} \vert ^{\alpha-1}}{b_{n}^{\alpha-1}}I \bigl( \vert X_{i} \vert \leq b_{n} \bigr) \\ &\quad \leq b_{n}^{1-\alpha} \vert a_{ni} \vert ^{\alpha}E \vert X_{i} \vert ^{\alpha}I \bigl( \vert X_{i} \vert \leq b_{n} \bigr) \\ &\quad \leq Cb_{n}^{1-\alpha} \vert a_{ni} \vert ^{\alpha}E \vert X \vert ^{\alpha}I \bigl( \vert X \vert \leq b_{n} \bigr) +Cb_{n} \vert a_{ni} \vert ^{\alpha}P \bigl( \vert X \vert >b_{n} \bigr) \\ &\quad \leq Cb_{n}^{1-\alpha} \vert a_{ni} \vert ^{\alpha}E \vert X \vert ^{\alpha}I \bigl( \vert X \vert \leq b_{n} \bigr) +Cb_{n}^{1-\alpha} \vert a_{ni} \vert ^{\alpha}E \vert X \vert ^{\alpha} \\ &\quad \leq Cb_{n}^{1-\alpha} \vert a_{ni} \vert ^{\alpha}E \biggl(\frac{ \vert X \vert ^{\alpha}}{ (\log(1+ \vert X \vert ) )^{\alpha/\gamma-1}}\cdot \bigl(\log\bigl(1+ \vert X \vert \bigr) \bigr)^{\alpha/\gamma-1} \biggr) \\ &\quad \leq Cn^{-1+1/\alpha} (\log{n} )^{1/\gamma-1} \vert a_{ni} \vert ^{\alpha}, \end{aligned}$$

we have

3.33 $$\begin{aligned} b_{n}^{-1}\sum_{i=1}^{n}E \vert a_{ni}X_{i} \vert I \bigl( \vert a_{ni}X_{i} \vert >b_{n}, \vert X_{i} \vert >b_{n} \bigr) \leq& Cb_{n}^{-1}n^{-1+1/\alpha} (\log{n} )^{1/\gamma-1}\sum_{i=1}^{n} \vert a_{ni} \vert \\ \leq& C (\log{n} )^{-1}\rightarrow0,\quad \text{as } n\rightarrow \infty \end{aligned}$$

and

3.34 $$\begin{aligned} b_{n}^{-1}\sum_{i=1}^{n}E \vert a_{ni}X_{i} \vert I \bigl( \vert a_{ni}X_{i} \vert >b_{n}, \vert X_{i} \vert \leq b_{n} \bigr) \leq& Cb_{n}^{-1}n^{-1+1/\alpha} (\log{n} )^{1/\gamma-1}\sum_{i=1}^{n} \vert a_{ni} \vert ^{\alpha} \\ \leq& C (\log{n} )^{-1}\rightarrow0,\quad \text{as } n\rightarrow \infty. \end{aligned}$$

Thus, (3.29) holds by (3.30)-(3.34). Therefore, we only need to prove that

3.35 $$\begin{aligned} \sum_{n=1}^{\infty}\frac{1}{n}P \Biggl( \max_{1\leq j\leq n} \Biggl\vert \sum_{i=1}^{j} (Z_{i}-EZ_{i} ) \Biggr\vert >\varepsilon b_{n} \Biggr)< \infty. \end{aligned}$$

Actually, by the Markov inequality, Lemma 2.2, Lemma 2.5, (3.18) and (3.28), we get

3.36 $$\begin{aligned} &\sum_{n=1}^{\infty}\frac{1}{n}P \Biggl( \max_{1\leq j\leq n} \Biggl\vert \sum_{i=1}^{j} (Z_{i}-EZ_{i} ) \Biggr\vert >\varepsilon b_{n} \Biggr). \\ &\quad \leq C\sum_{n=1}^{\infty}n^{-1}b_{n}^{-2} \sum_{i=1}^{n}E (Z_{i}-EZ_{i} )^{2} \\ &\quad \leq C\sum_{n=1}^{\infty}n^{-1}b_{n}^{-2} \sum_{i=1}^{n} E \vert a_{ni}X_{i} \vert ^{2}I \bigl( \vert a_{ni}X_{i} \vert \leq b_{n} \bigr) \\ &\quad \leq C\sum_{n=1}^{\infty}n^{-1}b_{n}^{-2} \sum_{i=1}^{n} E \vert a_{ni}X \vert ^{2}I \bigl( \vert a_{ni}X \vert \leq b_{n} \bigr) +C\sum_{n=1}^{\infty}n^{-1} \sum_{i=1}^{n}P \bigl( \vert a_{ni}X \vert >b_{n} \bigr) \\ &\quad \leq C\sum_{n=1}^{\infty}n^{-1}b_{n}^{-2} \sum_{i=1}^{n} E \bigl( \vert a_{ni}X \vert ^{\alpha}\cdot \vert a_{ni}X \vert ^{2-\alpha} \bigr)I \bigl( \vert a_{ni}X \vert \leq b_{n} \bigr)+C \\ &\quad \leq C\sum_{n=1}^{\infty}n^{-1}b_{n}^{-\alpha} \sum_{i=1}^{n} E \vert a_{ni}X \vert ^{\alpha}I \bigl( \vert a_{ni}X \vert \leq b_{n} \bigr)< \infty. \end{aligned}$$

Thus, the proof of Theorem 3.2 is completed. □

## Conclusions

In this paper, we use different methods from those of Sung [2] and Chen and Sung [3] to prove the results, and we obtain some strong convergence results for weighted sums of NSD random variables without the assumption of an identical distribution. Our results extend and improve the corresponding ones of Cai [1] and Sung [2] and Chen and Sung [3] to the case of NSD random variables.
